# Tailoring Pore Size and Surface Charge of Polyamide Reverse Osmosis Membranes via Alkaline Post-Treatment for Brackish Water Desalination

**DOI:** 10.3390/polym18080995

**Published:** 2026-04-19

**Authors:** Ying Li, Renzhong Wang, Zheng Liu, Yang Zhao, Long Li, Qian Cao, Feng Shao

**Affiliations:** 1 State Key Laboratory of Materials-Oriented Chemical Engineering, College of Chemical Engineering, Nanjing Tech University, Nanjing 211816, China; 202361104028@njtech.edu.cn (Y.L.);; 2 Suzhou National Laboratory, Suzhou 215123, China

**Keywords:** alkaline post-treatment, polyamide reverse osmosis membrane, pore size enlargement, surface charge enhancement, brackish water desalination

## Abstract

Overcoming the inherent permeability–selectivity trade−off is essential to broaden the practical application of polyamide (PA) reverse osmosis (RO) membranes in brackish water desalination. In this study, we developed a facile and cost-effective alkaline (NaOH) post-treatment method to fabricate high−performance loose-structured RO membranes. The NaOH post−treatment hydrolyzed part of the amide bonds within the membrane, converting them to negatively charged carboxyl groups. This process led to a slight increase in pore size and the formation of a looser structure. Molecular weight cut−off (MWCO) measurements confirmed that the pore size slightly increased from 0.19 nm to 0.21 nm, while X−ray photoelectron spectroscopy (XPS) and zeta potential measurements confirmed the conversion of amide bonds to carboxyl groups, which further enhanced the surface electronegativity. The synergistic effects of pore size enlargement and surface charge modification were elucidated as the key mechanisms for performance enhancement. The TPA membrane exhibited a 2−fold increase in water permeance (from 1.05 to 3.21 L m^−2^ h^−1^ bar^−1^), while the enhanced surface negative charge contributed to maintaining a high NaCl rejection of 98.5%. Additionally, the membrane also exhibited excellent pH stability as well as long-term stability over 100 h of continuous operation. This easily scalable post−treatment strategy offers a low−cost route to fabricate loose-structured membranes, with significant potential to enhance efficiency and reduce costs in brackish water desalination.

## 1. Introduction

The escalating global water scarcity, which currently affects over two billion people, has intensified the demand for advanced desalination technologies. Traditional thermal desalination technologies, such as multi−stage flash (MSF) and multi−effect distillation (MED), are well−established but suffer from drawbacks such as high energy consumption (All abbreviations are shown in Table A1). In recent years, membrane−based separation technologies have emerged as the mainstream approach for desalination due to their relatively low energy consumption and high modularity. Currently, a diverse array of membrane separation technologies suitable for desalination has been developed, each offering distinct mechanisms and performance characteristics tailored to specific applications [1]. Among these technologies, electrodialysis enables salt conversion and acid/base recovery without the need for chemical additives by dissociating water molecules under an electric field [2]. Nanofiltration (NF) membranes can selectively remove divalent ions in pretreatment or salt fractionation processes. Reverse osmosis (RO) membranes, as the dominant membrane technology for desalination, have achieved large−scale commercial adoption worldwide owing to their high salt rejection and low energy consumption [3,4]. Membrane performance is the key factor determining the overall cost−effectiveness of seawater desalination systems. Therefore, further enhancing the water permeability and antifouling properties of RO membranes is of great significance for reducing desalination costs and advancing membrane technology development. However, a primary obstacle to the wider application of RO membranes is the inherent permeability−selectivity trade−off [5], which makes it challenging to fabricate membranes that simultaneously achieve high selectivity and high water flux. This trade−off originates from the intrinsic structure of the PA thin film: an ultra−thin film shortens the water transport path [6,7], but a dense structure with underdeveloped water channels limits water permeance; conversely, a loose structure creates additional low−resistance pathways [8,9] yet compromises selectivity due to enlarged pore sizes. Membrane thickness and pore structure are key factors governing selectivity and water permeability, and optimizing both parameters is critical for enhancing overall membrane separation performance. Thus, significantly improving water permeability while maintaining high salt selectivity remains a challenging topic in RO membrane research.

Optimizing the membrane structure is an effective strategy to fabricate high−performance PA membranes [10,11], which is crucial for reducing operational and investment costs in desalination applications. Currently, various approaches have been developed to tailor the membrane structure, including: (i) incorporation of additives [12,13,14,15,16]; (ii) modification of the support layer [17,18,19,20]; (iii) selection of different monomers [21,22,23] or solvents [24,25,26]; and (iv) post-synthesis treatments [27,28,29,30,31]. Among these, post−treatment has attracted particular interest due to its simplicity and scalability. Notably, this technique is independent of the interfacial polymerization process and can enhance water permeability by precisely modifying the microstructure of the PA separation layer, making it more suitable for scale−up compared to other complex modification methods.

Among post−treatment strategies, constructing a loose membrane structure via post-processing is a viable approach to overcome the permeability–selectivity trade−off and obtain superior−performing PA membranes. Currently, the formation of loose PA membrane structures is mainly achieved through two routes: doping [32,33] and post−hydrolysis [34,35]. For instance, graphene [8,36,37] and metal–organic frameworks (MOFs) [38] have been incorporated into PA membranes to construct loose structures, thereby achieving excellent water permeability. However, these doping methods suffer from high costs and relatively complex preparation procedures, limiting their practical applications. In contrast, post−hydrolysis involves soaking the as−prepared membrane in a specific solution to induce controlled hydrolysis, thereby constructing a loose membrane structure. The resulting loose pores facilitate enhanced water permeability, while the changes in surface charge induced by hydrolysis can also partially strengthen the electrostatic repulsion effect (Donnan effect) [39], which is beneficial for maintaining high salt rejection. Generally, post−hydrolysis methods can be divided into two categories: one involves introducing hydrolyzable substances into the membrane interlayer, which are then hydrolyzed in situ within the membrane [40]; the other directly hydrolyzes polymer molecules in the PA separation layer using oxidative, acidic, or alkaline solutions [41,42,43]. After immersion in post−treatment reagents, the membrane structure undergoes controllable changes that adjust the pore size and further regulate water permeability. Additionally, the post−hydrolysis process enables more controllable and convenient fabrication of the desired loose structure, and it has been successfully validated in commercial PA membranes [44], highlighting its practical application potential.

To overcome the limitations of existing post−treatment methods (e.g., high cost, complex procedures), this study employs NaOH post−treatment to fabricate loose−structured PA RO membranes with enhanced water permeance. NaOH hydrolysis partially converts amide bonds into carboxyl groups, enhancing membrane performance. The effects of NaOH concentration and treatment time on membrane microstructure, physicochemical properties, and desalination performance were systematically investigated. After alkaline post−treatment, the membrane pore size increased from 0.19 nm to 0.21 nm, while surface hydrophilicity and electronegativity were significantly enhanced—all of which contributed to the improved overall membrane performance. The TPA membrane exhibited a water permeability approximately twice that of the original membrane (increasing from 1.05 to 3.21 L m^−2^ h^−1^ bar^−1^). Enhancing the water permeability of the membrane is of significant practical importance, as it allows for higher water flux at a given operating pressure or reduces the required operating pressure for a given water flux, potentially lowering energy consumption and saving costs. For brackish water reverse osmosis (BWRO), the upper limit of water permeability has been reported to be approximately 9 L m^−2^ h^−1^ bar^−1^ [45]. Alongside the increased permeability, the enhanced surface electronegativity of the membrane strengthened the Donnan exclusion effect, enabling the TPA membrane to maintain a NaCl rejection rate of up to 98.5%. Additionally, the TPA membrane demonstrated excellent pH stability and long−term operational durability over 100 h of continuous operation. This facile and cost−effective alkaline post−treatment offers a scalable strategy for fabricating loose−structured PA membranes, with significant potential for enhancing efficiency and reducing costs in brackish water desalination.

## 2. Experimental

### 2.1. Materials

Polysulfone (PSF) microporous membranes (Beijing Origin Water Technology Co., Ltd., (Beijing, China) were used as the support layer. The membranes, comprising a PSF coating on a polyester nonwoven fabric, were stored in a 2 wt% sodium bisulfite solution to prevent oxidation and microbial growth. Meta-phenylenediamine (MPD, ≥99%) was obtained from Sigma-Aldrich (St. Lous, MO, USA). Trimesoyl chloride (TMC, 98%), D(+)-10-camphorsulfonic acid|(+) (CSA, 98%), and triethylamine (TEA, 99%) were purchased from TCI Development Co., Ltd. (Shanghai, China). n-Hexane (analytical grade, ≥97%), sodium hydroxide (NaOH, ≥98%), potassium hydroxide (KOH, 95%), methanol (99.8%), ethanol (95%), ethylene glycol (99.5%), and polyethylene glycol (MW 300 and 400) were obtained from Greagent (Shanghai, China). Sodium chloride (NaCl, 99.5%) and triethylene glycol (TEG, 99.5%) were purchased from Adamas (Shanghai, China). Hydrochloric acid (HCl, 36–38%) was obtained from Yonghua Chemical Co., Ltd. (Suzhou, China). MPD was stored in light-protected containers. TMC and TEA were refrigerated at 0–8 °C. CSA was kept in a desiccator. Other chemicals were stored under ambient conditions in chemical cabinets. All materials were used as received, without any further purification. Deionized water (resistivity of 18.2 MΩ·cm) used in all experiments was prepared using an ultrapure water system (Miaozhiyi Electronic Technology Co., Ltd., Nanjing, China).

### 2.2. Preparation of the Composite Membranes

**Figure 1:**
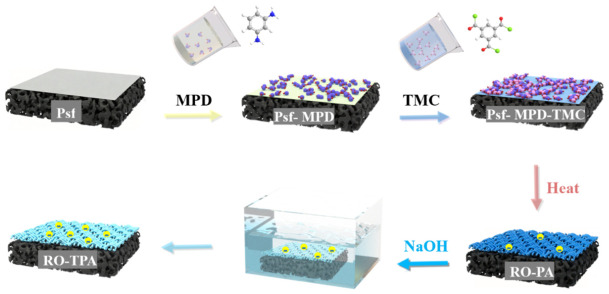
TPA Reverse Osmosis Membrane Fabrication Flowchart.

The PA membrane was prepared using the classical interfacial polymerization (IP) method [46]. Prior to membrane fabrication, an aqueous solution containing 2.5 wt% MPD monomer and an organic solution containing 0.25 wt% TMC monomer were prepared (for detailed TMC concentration optimization, see Figure S8). Both solutions were ultrasonicated for 5 min to ensure complete dissolution and uniform dispersion of the monomers. The PSF support membrane was first fixed onto a PTFE hollow frame using solvent−resistant double−sided tape. Then, 30 mL of the aqueous solution was poured onto the membrane surface (effective area of 36 cm^2^) and allowed to soak for 30 s. After pouring off the excess solution, the membrane was dried until the surface appeared matte, indicating the removal of visible water droplets. Next, the organic solution was poured onto the MPD−saturated membrane surface and allowed to react for 30 s, after which the excess solution was drained. Once the membrane surface was dried, it was placed in a hot air oven at 70 °C for 5 min to evaporate the organic solvent and promote further cross−linking of the PA layer. Finally, the membrane was removed from the oven, cooled to room temperature for 10 min, and then thoroughly rinsed with deionized water to remove unreacted monomers. After soaking in deionized water for 1 h to remove residual chemicals and stabilize the membrane, the PA membrane was subjected to alkaline post−treatment using NaOH solutions under various conditions (concentration and time). The treated membrane was then rinsed with copious deionized water to remove any residual NaOH, resulting in the TPA membrane (as shown in Figure 1). To identify the optimal post−treatment conditions, a series of NaOH concentrations and treatment times were investigated, as summarized in Table 1. Membranes intended for performance evaluation were stored in a 0.5 wt% NaHSO_3_ solution at 4 °C to prevent microbial growth, while membranes for characterization were stored in deionized water at room temperature.

### 2.3. Characterization of the PA Membranes

Before characterization, the membrane was dried in a vacuum oven at 40 °C for 12 h to remove moisture without changing its morphology. The surface and cross−sectional morphology of the membrane were measured using a Zeiss Sigma 300 scanning electron microscope (SEM). Prior to observation, samples were sputter−coated with a thin layer of gold (Au) using a SuPro Instruments Mini Coater. Energy−dispersive spectroscopy (EDS) was also used to observe the distribution of different elements on the surface of the samples. The thickness of the film was obtained through transmission electron microscopy (TEM, JEM−2100F, Tokyo, Japan). Before TEM testing, the samples were embedded, sectioned, and transferred to copper grids (with carbon−coated support films). Scanning was conducted using a 200 kV accelerating voltage. The surface roughness of the membrane was obtained by scanning the membrane surface with an atomic force microscope (AFM; NTEGRA Spectra II, NT−MDT). The collected data were processed with gwyddion software to generate the final images. Surface roughness was assessed using the average roughness (Ra) and root mean square roughness (Rq). The chemical functional groups of the PA membranes were characterized using attenuated total reflectance Fourier transform infrared (ATR−FTIR) spectroscopy (Thermo Fisher Scientific Nicolet iS20). Data were automatically smoothed and baseline−corrected using omnic software. For X−ray photoelectron spectroscopy (XPS) analysis, a Thermo Scientific K−Alpha XPS system was used. The excitation source was Al Kα X−ray with a working voltage of 12 kV and 6 mA. Spectral fitting and elemental quantification were performed using avantage software. The zeta potential and water contact angle of the composite membranes were measured using an electrokinetic analyzer (SurPASS 3, Graz, Austria) and a contact angle goniometer (Attension Theta Flex, Biolin Scientific, Gothenburg, Sweden), respectively.

### 2.4. MWCO and Membrane Pore Distribution

The typical molecular weight cutoff (MWCO) of reverse osmosis membranes is usually 100–200 Da. In this study, six neutral solutes were used to determine the MWCO and pore size distribution, covering a continuous molecular weight range from 32 Da to 400 Da (as shown in Table 2). The feed solution contained 200 mg/L of each neutral solute. The system was operated at a pressure of 15.5 bar, a temperature of 25 ± 1 °C. The rejection rate was calculated from the contents of PEGs in the feed and filtrate solutions, as obtained by a total organic carbon analyzer (Xpert−TOC−TNb, TE instruments, Delft, The Netherlands). The rejection rate was calculated according to Equation (1). Subsequently, a fitting curve was obtained based on the molecular weights of the neutral solutes and their corresponding rejection rates. The MWCO of the membrane is defined as the molecular weight of the solute at which the rejection exceeds 90%. The relationship between the Stokes radius *r_s_* of neutral solutes and molecular weight can be expressed by Equation (2) [47]. Based on the molecular weights corresponding to the rejection rates of 84.13% and 50% in the fitting curve, combined with Equation (2), the constants *µ_p_* and *σ_p_* can be determined. Then, the pore size distribution of the membrane can be obtained from the plot according to Equation (3) [48].

(1) $$R=\left(1-\frac{C_{g}}{C_{h}}\right)\times 100\%$$

where *C_g_, C_h_*, and *R* correspond to the permeate solution concentration (mg/L), the feed solution concentration (mg/L), and the rejection rate of the target solute (%), respectively.

(2) $$\log r_{s}=-1.3363+0.395\log Mw$$

(3) $$\frac{dR\left(r_{p}\right)}{dr_{p}}=\frac{1}{r_{p}\ln\sigma_{P}\sqrt{2\pi }}\exp\left[-\frac{{\left(\ln r_{P}-\ln\mu_{P}\right)}^{2}}{2{\left(\ln\sigma_{p}\right)}^{2}}\right]$$

where *r_s_* and *Mw* are the Stokes radius (nm) and molecular weight (Da) of a neutral solute, respectively; *r_p_* is the pore size of the membrane (nm); *µ_p_* represents the mean pore size of the membrane, corresponding to the Stokes radius (nm) of a solute with a 50% rejection rate; and *σ_p_* represents the ratio of the radius of a solute with an 84.13% rejection rate to the radius of a solute with a 50.00% rejection rate.

### 2.5. Separation Performance Measurements

The separation performance of the composite membrane was evaluated using a cross-flow filtration device with a separation area of 21 cm^2^ (4.2 cm × 5 cm). The test conditions were a temperature of 25 ± 1 °C and a feed flow rate of 1 L/min. A 2000 mg/L NaCl solution was used as the feed to simulate brackish water conditions. Each membrane was pre-compacted at 15.5 bar for 2 h until achieve stable performance. Subsequently, a 1 h rejection test was conducted under steady-state conditions. Permeate and feed samples were collected, and their conductivities were measured using a calibrated conductivity meter (model, manufacturer) to calculate salt rejection. All data were obtained as averages from three independently prepared membrane samples. Permeability (*A*, L m^−2^ h^−1^ bar^−1^) and salt rejection (*R*, %) were calculated using the following formulas.

(4) $$A=\frac{\Delta V}{S\Delta T\Delta P}$$

(5) $$R=\left(1-\frac{C_{P}}{C_{f}}\right)\times 100\%$$

where *V*(L) is the permeate volume, *S*(m^2^) is the membrane filtration area, Δ*T*(h) is the permeation time, and Δ*P*(bar) is the trans-membrane pressure. *C_f_* and *C_p_* are the conductivities of the feed and filtrate, respectively, measured using a conductivity meter (HQ4200, Hach, Loveland, CO, USA). All measurements were performed three times and averaged.

## 3. Results and Discussion

### 3.1. Physical Structure of Membranes

**Figure 2:**
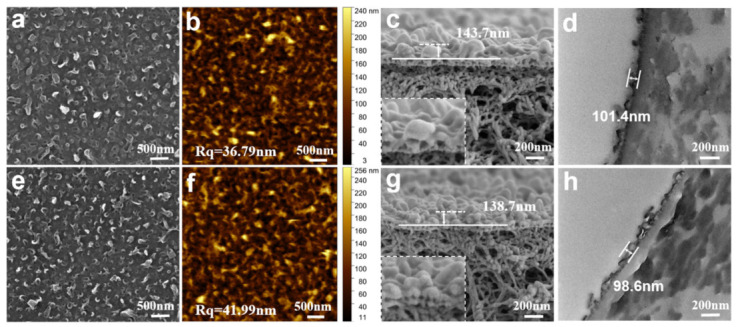
Microscopic morphology of the films. (**a**) SEM image of the PA membrane surface. (**b**) AFM image of the PA membrane surface. (**c**) Cross−sectional SEM image of the PA membrane. (**d**) Cross−sectional TEM image of the PA membrane. (**e**) SEM image of the TPA membrane surface. (**f**) AFM image of the TPA membrane surface. (**g**) Cross−sectional SEM image of the TPA membrane. (**h**) Cross−sectional TEM image of the TPA membrane.

The surface microstructures of the PA and TPA (soaked for 2 days in a pH 13 environment) films were observed using SEM and AFM. As shown in Figure 2a,e, both the pristine PA and TPA membranes exhibited the typical ridge−valley structure characteristic of polyamide films, which was uniformly distributed across the surface, in contrast to the smooth surface of the polysulfone support (Figure S1). The surface morphology of the membrane does not change significantly before and after alkaline treatment. AFM images (Figure 2b,f) corroborated the SEM observations, revealing minimal topographic changes after alkaline treatment. The surface roughness of the films was further quantified using AFM images. The treated TPA membranes showed a slightly higher root mean square roughness (Rq = 41.99 nm) compared to the pristine PA membranes (Rq = 36.79 nm). This marginal increase may be attributed to the partial cleavage of amide bonds on the membrane surface during alkaline hydrolysis. Studies have indicated that the surface roughness of a membrane is related to its water permeability [49]. SEM and TEM were used to characterize the cross-sections of the films. SEM images of the film cross −sections were shown in Figure 2c,g. The cross−sectional SEM images indicated that both films exhibit similar ridge−and−valley structures. The separation layer thickness of the PA film was approximately 143.7 nm, while the separation layer thickness of the TPA film was around 138.7 nm, suggested that alkaline treatment did not significantly alter the film thickness. Consistent with the surface morphology, the film surfaces were uniformly dense. More detailed high−resolution transmission electron microscopy (TEM) images (Figure 2d,h) showed that the polyamide layer consisted of dark regions of dense layers and bright regions corresponding to nanobubbles. The thickness of the PA membrane separation layer was around 101.4 nm, while the TPA membrane separation layer was approximately 98.6 nm, indicating that the thickness difference between the two was not significant. This was consistent with the results obtained from SEM cross−section tests. It was worth noting that due to differences in sample preparation methods for SEM and TEM testing, discrepancies existed between the results obtained by the two techniques. The AFM 3D height distribution (Figure S2) shows ridge−valley peaks on the film surface, corresponding to the larger leaf−like folds observed in SEM images. In summary, SEM, TEM, and AFM analyses revealed that alkaline treatment did not significantly alter the surface morphology, roughness, or thickness of the polyamide layer. The treated membranes retained the characteristic ridge−valley structure with only a marginal increase in surface roughness. Overall, in terms of microstructure, the alkali treatment has a minimal impact on the films.

### 3.2. Alkaline Hydrolysis Mechanism of Membranes

**Figure 3:**
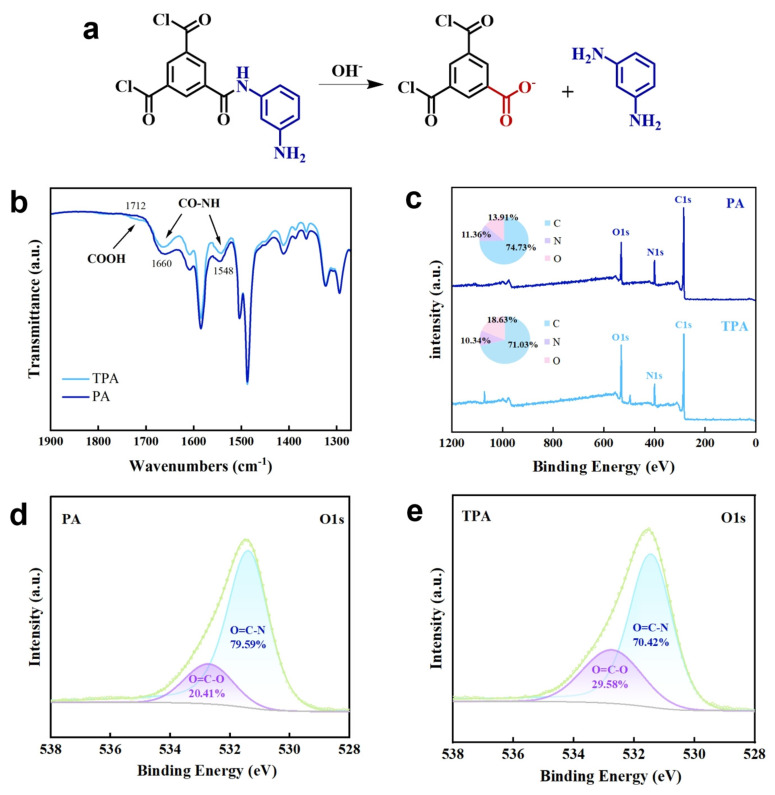
Alkaline hydrolysis mechanism of the films. (**a**) Hydrolysis process of the amide bond in alkaline solution. (**b**) FTIR spectra of PA and TPA films. (**c**) XPS survey spectra of PA and TPA films. (**d**,**e**) High−resolution O1s spectra of PA and TPA films.

The amide bonds in polyamide membranes possess inherent chemical stability due to the resonance between the lone pair electrons of nitrogen and the carbonyl oxygen. However, under highly alkaline conditions [42], this resonance structure can be disrupted by nucleophilic attack of OH^−^ (Figure 3a). Specifically, the alkaline hydrolysis of polyamide membranes typically occurred through nucleophilic attack by hydroxide ions on the carbon of the amide carbonyl group, with the rate−determining step being the formation of a tetrahedral intermediate. The hydrolysis process included both the cleavage of amide bonds that led to main chain scission of the polymer and the transformation of amide groups into side−chain carboxyl groups without causing main chain breakage. These two pathways collectively altered the physicochemical structure of the membrane: main chain scission reduced crosslinking and weakened the skeleton of the membrane separation layer, while side−chain carboxylation enhanced surface hydrophilicity and electrostatic repulsion. It was noteworthy that the polysulfone support layer, serving as the main framework of the composite membrane, possessed excellent alkali resistance, providing reliable support for the polyamide separation layer. To elucidate the hydrolysis mechanism of the membrane, the chemical properties of the films before and after hydrolysis were compared. ATR-FTIR spectra of the pristine PA and treated TPA membranes are shown in Figure 3b. The spectrum of the pristine PA membrane exhibited characteristic peaks at 1712 cm^−1^ (O=C–OH stretching), 1660 cm^−1^ (amide I, C=O stretching), and 1548 cm^−1^ (amide II, N−H in-plane bending). Specifically, compared with the PA film, the TPA film shows a significant decrease in the C=O and N−H peaks, while the O=C–OH peak increases, indicating that partial the amide bonds of the PA film were hydrolyzed after alkaline post−treatment, exposing some carboxyl groups. XPS analysis further corroborated these findings. As shown in Figure 3c, after treatment, the nitrogen element content on the TPA film surface decreases, whereas the oxygen element content increases, which is consistent with the SEM-EDS analysis results (Figure S3). The N/O ratio could serve as a probe for the degree of surface cross−linking [43,50]; a higher N/O ratio generally indicated a denser film structure. The N/O ratio of TPA films decreased from 0.81 in the original PA to 0.56, suggesting a lower degree of film cross−linking (Table S1), which was consistent with the results from FTIR and changes in film performance. It should be noted that this method could only provide a semi−quantitative assessment of the degree of cross−linking, rather than absolute quantitative results. High−resolution C1s XPS spectra (Figure S4) revealed a decrease in the C−N component after alkaline treatment, consistent with amide bond hydrolysis. Quantitative analysis of the high-resolution O 1s spectra (Figure 3d,e) showed that after alkaline treatment, the relative area of the O=C–N component decreased from 79.6% to 70.4%, while the O=C–O component increased from 20.4% to 29.6%, confirming the structural changes of the membrane.

### 3.3. Properties of Membranes

**Figure 4:**
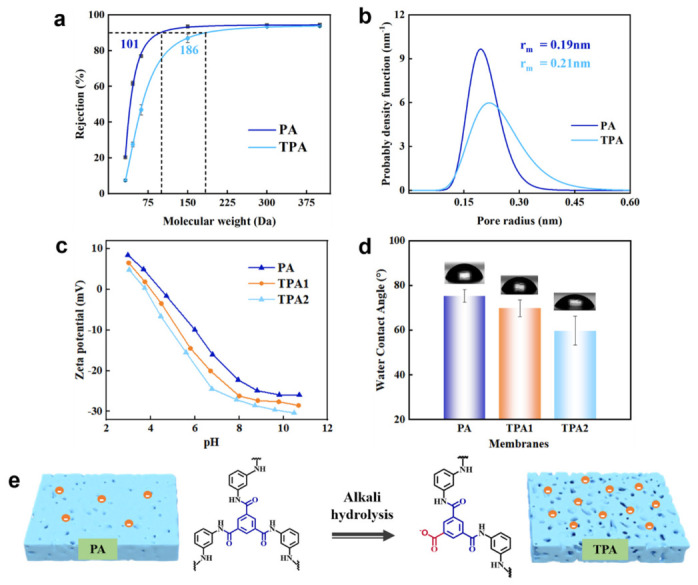
Characteristics of the original PA and TPA membranes. (**a**) MWCO of PA and TPA membranes. (**b**) Average pore size of PA and TPA membranes. (**c**) Zeta potential of PA and TPA12 composite membranes. (**d**) Water contact angle of PA and TPA12 composite membranes. (**e**) Structural diagrams of PA and TPA membranes. (PA membrane treated with base 13 for 1 day to obtain TPA1, PA membrane treated with base 13 for 2 days to obtain TPA2).

To elucidate the effects of alkaline treatment on membrane microstructure, we characterized the pore size, surface charge, and hydrophilicity of the pristine PA and TPA membranes. Pore size changes were evaluated by rejection tests using neutral organic molecules (Table S2) [51]. As shown in Figure 4a, the pristine PA membrane exhibited an MWCO of 101 Da, while the TPA membrane showed a larger MWCO of approximately 186 Da. This indicates that the effective pore size increased after alkaline treatment, confirming that the post−treatment loosened the membrane microstructure. In addition, the average pore size of the membrane was calculated based on the MWCO results, as shown in Figure 4b. After NaOH post−treatment, the membrane pore size increased from 0.19 nm to 0.21 nm. This pore size enlargement was attributed to the partial hydrolysis of amide bonds by NaOH, which contributed to the observed 2.01−fold increase in water permeance. We further investigated the hydrophilicity and charge characteristics of the PA membranes. As shown in Figure 4c, the TPA membrane exhibited stronger negative surface charge compared to the PA membrane. Notably, the feed solution employed in this study mimicked the actual brackish water environment, with a measured pH of approximately 6. Specifically, at pH 6, the zeta potentials of PA, TPA1, and TPA2 were measured to be −10.24 mV, −15.68 mV, and −18.62 mV, respectively. Based on the measured zeta potentials, we quantitatively estimated the Donnan potential (see Table S6 for details) and found that the absolute Donnan potential of the TPA2 membrane increased by approximately 39% compared to that of the PA membrane. The enhanced negative charge of TPA2 strengthened the Donnan exclusion effect [52], which helped the membrane maintain a high NaCl rejection rate even with increased pore size. As shown in Figure 4d, the water contact angles of PA, TPA1, and TPA2 were 75.0°, 69.8°, and 59.6°, respectively. This indicates that alkaline post−treatment rendered the membrane surface more hydrophilic, attributed to the increased content of carboxyl groups. The enhanced hydrophilicity contributed to the improved water permeance [53]. Based on these findings, a schematic illustration of the membrane structures is presented in Figure 4e. The pristine PA membrane had a thin, densely crosslinked structure. After NaOH treatment, partial hydrolysis of amide bonds generated carboxyl groups, leading to an increased pore size and a loosened membrane structure while preserving the thin film integrity [45].

### 3.4. Desalination Performance and Stability of the Membrane

**Figure 5:**
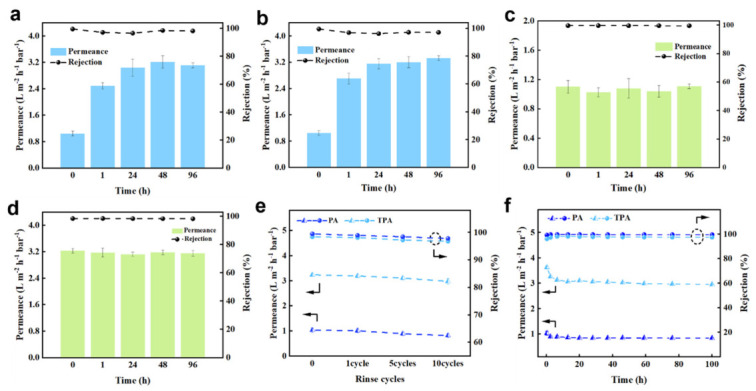
Permeation selectivity and stability of the membranes. (**a**) Variation of water permeation rate and rejection rate of TPA composite membranes with hydrolysis time (soaked in NaOH solution at pH = 13). (**b**) Variation of water permeation rate and rejection rate of TPA composite membranes with hydrolysis time (soaked in KOH solution at pH = 13). (**c**) Variation of water permeation rate and rejection rate of PA composite membranes with hydrolysis time (soaked in HCl solution at pH = 2). (**d**) Variation of water permeation rate and rejection rate of TPA composite membranes with hydrolysis time (soaked in HCl solution at pH = 2). (**e**) The change of membrane performance of the PA and TPA membranes after acidic/alkaline washing. (**f**) Long-term stability tests of the original PA and TPA membranes. Each data point is measured using three samples and the average is taken.

The separation performance of the TPA composite membranes was evaluated using a cross−flow filtration system (Figure 5). The effects of NaOH concentration (pH 12.5, 13, and 13.5) and soaking time on membrane performance were systematically investigated (Figure 5a, Figures S5 and S6). The results indicated that both NaOH concentration and soaking time influenced membrane performance, with pH 13 yielding the optimal overall performance. As shown in Figure 5a, with increasing soaking time in pH 13 NaOH solution, the water permeance of the TPA membrane increased from 16.3 to 49.8 L m^−2^ h^−1^ (at 15.5 bar). It is noteworthy that after 1 h of post−treatment, the water permeability of the TPA membrane can be significantly increased to 2.4 times that of the PA membrane, which demonstrates practical feasibility for potential industrial applications. The optimal treatment time for the membrane was 48 h, at which point the TPA membrane exhibited the best overall performance. Compared with the original PA membrane, the water flux was increased to three times (from 1.05 L m^−2^ h^−1^ bar^−1^ to 3.21 L m^−2^ h^−1^ bar^−1^), while maintaining a NaCl rejection rate of up to 98.5%. This post−treatment method was also applicable to polyamide membranes fabricated via the free−standing interfacial polymerization method (Figure S7). Owing to their ultrathin PA separation layer, these membranes exhibited enhanced performance within a shorter treatment time (Table S3). The effect of other alkalis was also examined using pH 13 KOH solution (Figure 5b). With increasing soaking time, the water permeance increased from 16.2 to 51.1 L m^−2^ h^−1^, while maintaining a high NaCl rejection. It is noteworthy that compared with membranes treated with NaOH, membranes treated with KOH exhibited slightly higher overall water permeability and slightly lower overall NaCl rejection. This could be attributed to the effect of K^+^. With a smaller hydrated radius, K^+^ could more easily penetrate the membrane layer, inducing stronger segmental relaxation and thus higher water flux. At the same time, K^+^ possessed lower hydration energy, which made it more effective at shielding the negative charges on the membrane surface, thereby weakening the Donnan effect and reducing the salt rejection of the membrane. The results indicated that the type of alkali (different cations) exerted a certain influence on membrane performance. However, compared with the changes in membrane performance obtained from different neutral cation solution treatments (Figure S9), which were far smaller than those of the TPA membrane obtained using alkali treatment, this indicated that OH^−^ played a dominant role in the changes in membrane performance.

Additionally, the performance variations of the PA and TPA membranes were investigated in a strongly acidic environment at pH 2 (Figure 5c,d). The results showed that the water permeability and salt rejection of both membranes remained largely stable, indicating that the strongly acidic environment exerted negligible effects on the membrane properties. Notably, acidic and alkaline cleaning protocols are commonly employed to remove foulants accumulated during membrane operation [54]. Accordingly, we evaluated the separation performance of the membranes after multiple cleaning cycles (Figure 5e). After 10 acid-base cleaning cycles, the salt rejection of the TPA membrane decreased by 1.6%, while its water permeability remained stable at 2.98 L m^−2^ h^−1^ bar^−1^, demonstrating that the TPA membrane exhibited excellent pH stability. The salt rejection of the pristine PA membrane decreased by 1.7%. To investigate the effect of alkaline treatment on membrane structure, we measured the mechanical properties of the PA membrane and the TPA membrane (Figure S10). The results showed that the tensile strength and elongation at break of the TPA membrane were generally comparable to those of the PA membrane. In practical applications, the long−term operational stability of TPA composite membranes is crucial. As shown in Figure 5f, a 100 h continuous brackish water desalination test was conducted on the PA and TPA membranes. Both water permeance and salt rejection remained stable throughout the test, indicating good operational stability and structural integrity. Specifically, during the first 2 h of operation after the membrane reaches steady-state conditions, the water flux slightly decreased from 3.17 to 3.03 L m^−2^ h^−1^ bar^−1^, and then remained relatively stable. The salt rejection also reached a steady state within the same period. This initial decline in water permeance was attributed to the compaction of the PA support layer under pressure [55], which is known to affect membrane permeability. Overall, the TPA membranes fabricated via alkaline post−treatment exhibited superior separation performance and considerable potential for practical applications.

**Figure 6:**
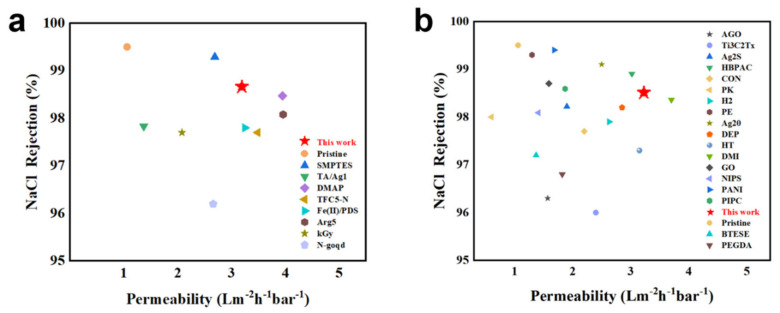
(**a**) Comparison of our membrane with recently reported post-treated reverse osmosis membranes. (**b**) Comparison of our membranes with recently reported advanced reverse osmosis membranes. Detailed information on the referenced RO membranes is provided in Table S4 [56,57,58,59,60,61,62,63] and Table S5 [16,50,64,65,66,67,68,69,70,71,72,73,74,75,76,77,78,79].

To further demonstrate the progress of this work, as shown in Figure 6a, we collected the membrane performance of other post-treatments reported in recent literature for comparison with the TPA membrane, clearly highlighting the advantages and competitiveness of our post−treatment strategy. We also gathered data from recent years on other methods used to improve membrane performance, not limited to post−treatment, as shown in Figure 6b. Based on an intuitive comparison, it was found that the water permeability−selectivity performance of TPA membranes is superior to that of most other RO membranes reported in the literature. These findings demonstrate that the thin and loose structure achieved by alkaline post−treatment offers a promising strategy for designing RO membranes with both high water permeance and excellent selectivity.

Despite the significant improvements in membrane performance achieved by the alkaline post-treatment, it should still be acknowledged that there are limitations. The membrane exhibited optimal performance after 48 h of post-treatment; however, for certain industrial processes, a 48 h post−treatment period may be relatively long. In line with the requirements of industrial production, a balance between high performance and high efficiency can be achieved by optimizing other parameters. In future process research, various parameters could be optimized to shorten the post−treatment time and further reduce time costs. For example, using an alkaline solution with a pH greater than 13 could achieve a multiple−fold increase in water permeability in a shorter period, while simultaneously introducing a large number of negative charges on the surface (through grafting or other feasible methods) to leverage stronger charge effects, thereby compensating for or even enhancing the NaCl rejection rate.

## 4. Conclusions

This study demonstrated a facile and cost−effective alkaline post-treatment strategy for fabricating high-permeance polyamide reverse osmosis membranes. Alkaline hydrolysis partially converted amide bonds within the membrane into carboxyl groups. Through the synergistic effects of enlarged pore size, enhanced hydrophilicity, and increased surface electronegativity, the TPA membrane achieved a water permeance of 3.21 L m^−2^ h^−1^ bar^−1^, a 2−fold increase compared to the pristine PA membrane (1.05 L m^−2^ h^−1^ bar^−1^), while maintaining a high NaCl rejection of 98.5%. The optimized TPA membrane exhibited a superior permeability–selectivity combination compared to most reported RO membranes, along with excellent pH stability and long-term operational stability over 100 h. Overall, this work systematically investigated the effects of alkaline hydrolysis on the pore structure, surface properties, and separation performance of polyamide membranes. The findings highlight the great potential of alkaline post−treated RO membranes for efficient and cost-effective brackish water desalination.

## Figures and Tables

**Table 1:** Alkalinity-Processing Time Parameter Table.

Membranes	Soaking Time (h)				
TPA−pH12.5	0	1	24	48	96
TPA−pH13	0	1	24	48	96
TPA−pH13.5	0	1	24	48	96

**Table 2:** Molecule weight of six different neutral solutions at 25 °C.

Neutral Solutions	Methanol	Ethanol	Ethylene Glycol	Triethylene Glycol	Polyethylene Glycol	
Molecule weight (Da)	32	46	62	150	300	400

## Data Availability

The original contributions presented in this study are included in the article/Supplementary Materials. Further inquiries can be directed to the corresponding author.

## Supplementary Materials

The following supporting information can be downloaded at: https://www.mdpi.com/article/10.3390/polym18080995/s1, Figure S1: SEM Image of Polysulfone (Psf) Based Membrane; Figure S2: AFM 3D Topography of the Membrane; Figure S3: EDS element mapping and analysis of the membrane; Figure S4: Deconvolution of the C1s of XPS membrane; Figure S5: TPA Membrane Gradient Post-Treatment Time—Performance Variation Chart (pH = 12.5); Figure S6: TPA Membrane Gradient Post-Treatment Time—Performance Variation Chart (pH = 13.5); Figure S7: Morphology and thickness of free-standing PA film; Figure S8: Effect of TMC Concentration on Membrane Performance; Figure S9: Effect of Post-Treatment with Na^+^ and K^+^ Solutions on PA; Figure S10: Tensile Stress-Strain Curves of PA and TPA; Table S1: The XPS results of the PA and TPA nanofilms; Table S2: Spherical neutral solutes with various molecular weights were selected for rejection tests; Table S3: Effect of alkaline post-treatment on PA membranes prepared by the free interface(FIP) method; Table S4: Performance of RO membranes prepared in this work and in the literature; Table S5: Performance of RO membranes prepared in this work and in the literature; Table S6: Surface charge density and Donnan potential calculated from zeta potentials measured in 1 mM KCl solution [7,16,50,56,57,58,59,60,61,62,63,64,65,66,67,68,69,70,71,72,73,74,75,76,77,78,79].

## Appendix A

**Table A1:** The list of abbreviations with their explanations.

Number	Abbreviation	Explanation
1	MSF	Multi-stage flash
2	MED	Multi-effect distillation
3	NF	Nanofiltration
4	IP	Interfacial polymerization
5	PA	Polyamide
6	TPA	Treated polyamide
7	RO	Reverse osmosis
8	PSF	Polysulfone
9	MPD	Meta-phenylenediamine
10	TMC	Trimesoyl chloride
11	CSA	D(+)-10-camphorsulfonic acid|(+)
12	TEA	Triethylamine
13	TEG	Triethylene glycol
14	NaOH	Sodium Hydroxide
15	KOH	Potassium Hydroxide
16	NaCl	Sodium chloride
17	HCl	Hydrochloric Acid
18	NaHSO_3_	Sodium Bisulfite
19	MWCO	Molecular weight cutoff
20	TOC	Total organic carbon
21	SEM	Scanning electron microscope
22	EDS	Energy-dispersive spectroscopy
23	TEM	Transmission electron microscopy
24	AFM	Atomic force microscope
25	Rq	Root mean square roughness
26	Ra	Average roughness
27	Da	Dalton
28	XPS	X-ray photoelectron spectroscopy
29	ATR-FTIR	Attenuated total reflectance Fourier transform infrared
