# Supplementary material for: Tailoring Pore Size and Surface Charge of Polyamide Reverse Osmosis Membranes via Alkaline Post-Treatment for Brackish Water Desalination
Source: Polymers (Basel). 2026 Apr 19;18(8):995. doi: 10.3390/polym18080995 (PMC13120458; doi:10.3390/polym18080995)
Supplement: Supplementary file 1 [file polymers-18-00995-s001.zip › polymers-4223650-supplementary.pdf]

Supporting Information

Tailoring Pore Size and Surface Charge of Polyamide Reverse Osmosis Membranes via Alkaline Post-Treatment for Brackish Water Desalination

Ying Li[1, 2], Renzhong Wang[1, 2], Zheng Liu[1, 2], Yang Zhao[1, 2], Long Li[1, 2], Qian Cao[1, 2], Feng Shao[1, 2]*

1. State Key Laboratory of Materials-Oriented Chemical Engineering, College of Chemical Engineering, Nanjing Tech University, Nanjing 211816, China;

2. Suzhou National Laboratory, Suzhou 215123, China.

** Correspondence: feng.shao@njtech.edu.cn.*

Table of contents

1. Supporting Figures

Figure S1. SEM Image of Polysulfone (Psf) Based Membrane.

Figure S2. AFM 3D Topography of the Membrane.

Figure S3.EDS element mapping and analysis of the membrane.

Figure S4.Deconvolution of the C1s of XPS membrane.

Figure S5.TPA Membrane Gradient Post-Treatment Time - Performance Variation Chart (pH=12.5).

Figure S6.TPA Membrane Gradient Post-Treatment Time - Performance Variation Chart (pH=13.5).

Figure S7. Morphology and thickness of free-standing PA film.

Figure S8. Effect of TMC Concentration on Membrane Performance.

Figure S9. Effect of Post-Treatment with Na^+ and K^+ Solutions on PA.

Figure S10. Tensile Stress-Strain Curves of PA and TPA.

Table S1. The XPS results of the PA and TPA nanofilms.

Table S2. Spherical neutral solutes with various molecular weights were selected for rejection tests.

Table S3. Effect of alkaline post-treatment on PA membranes prepared by the free interface(FIP) method.

Table S4. Performance of RO membranes prepared in this work and in the literature.

Table S5. Performance of RO membranes prepared in this work and in the literature.

Table S6. Surface charge density and Donnan potential calculated from zeta potentials measured in 1 mM KCl solution.

2.Supporting References

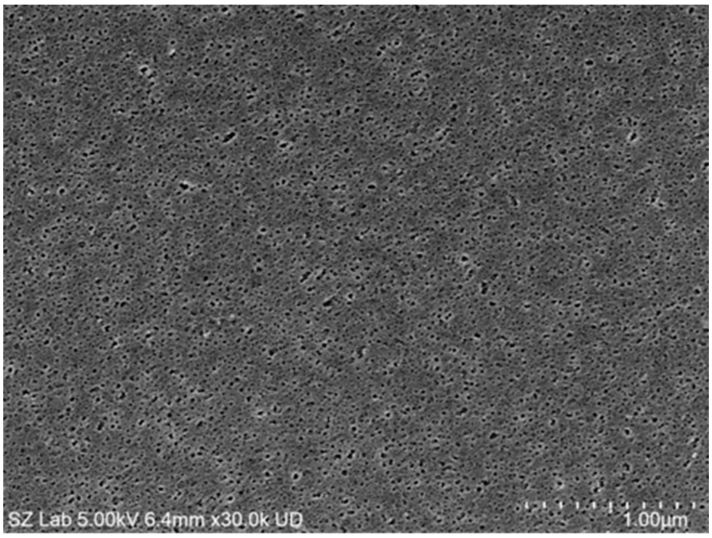

Fig. S1. SEM Image of Polysulfone (Psf) Based Membrane.

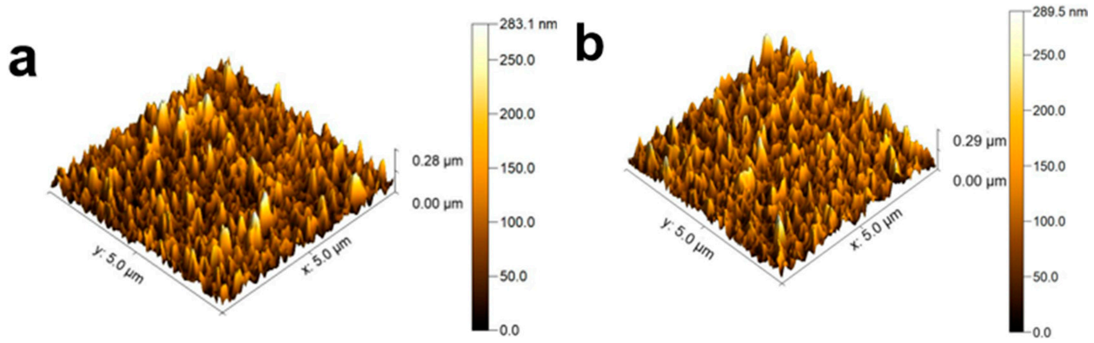

Fig. S2. AFM 3D Topography of the Membrane. a）3D Topographic Image of PA Membrane by AFM.　b）3D Topographic Image of TPA Membrane by AFM.

The surface height distribution of the membrane remained essentially unchanged after alkaline treatment, consistent with the cross-sectional SEM and TEM images.

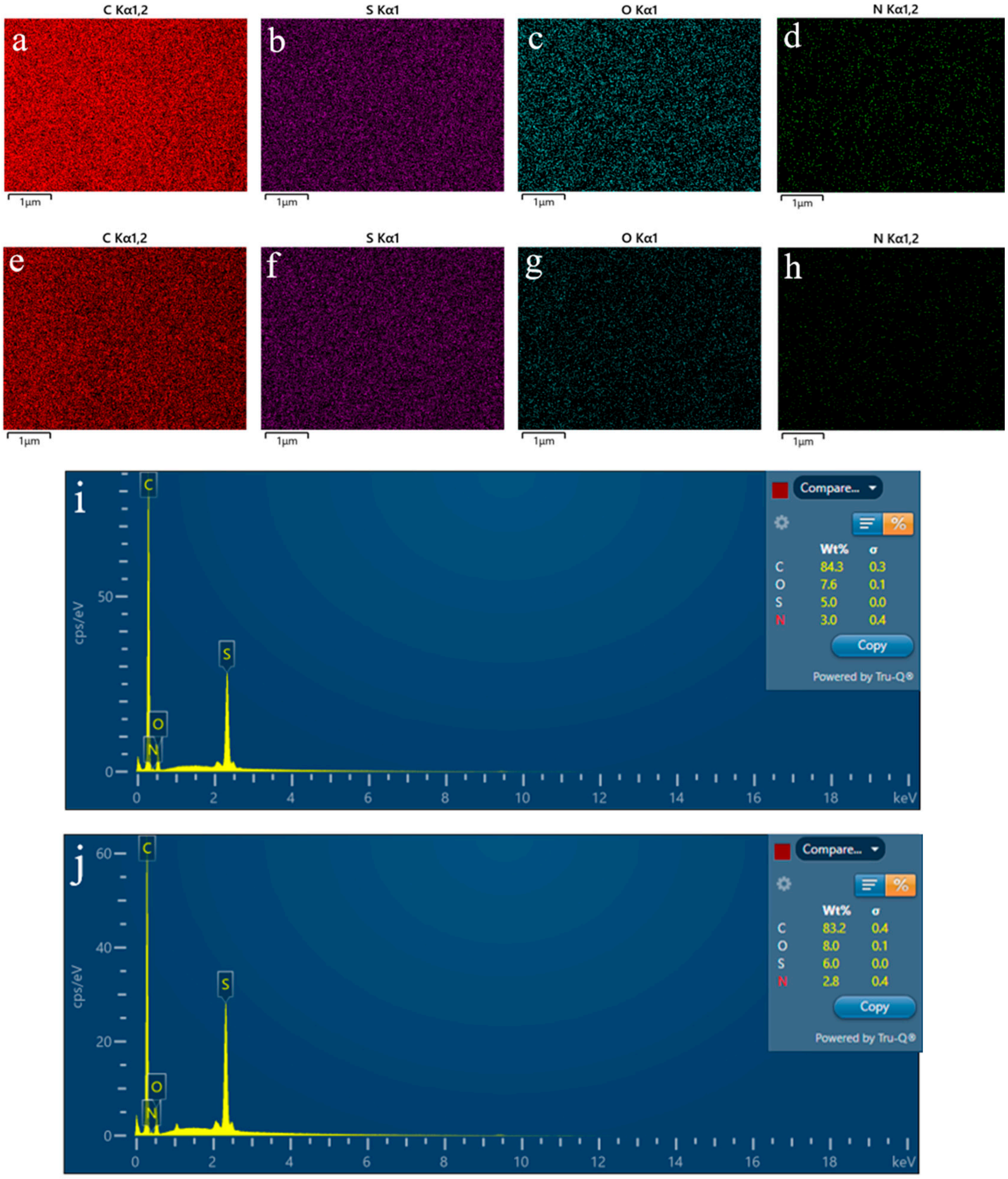

Fig. S3.EDS element mapping and analysis of the membrane. (a-d)EDS Elemental Analysis and Imaging of PA Membrane. (e-h)EDS Elemental Analysis and Imaging of TPA Membrane. (i)Analysis of the C, S, O, and N Elemental Composition of PA Membrane. (j)Analysis of the C, S, O, and N Elemental Composition of TPA Membrane.

EDS analysis showed that the oxygen content increased from 7.6% to 8.0% while the nitrogen content decreased from 3.0% to 2.8% after alkaline treatment, confirming the partial hydrolysis of amide bonds.

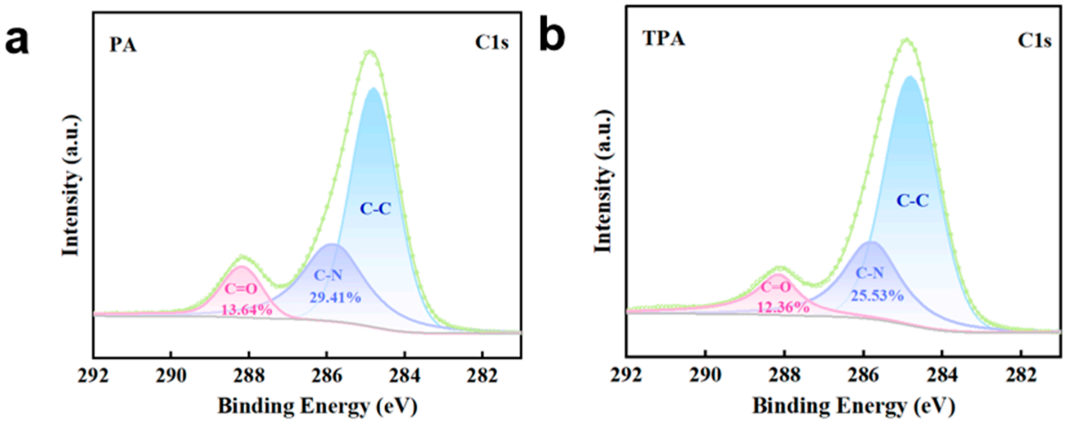

Fig. S4. Deconvolution of the C1s of XPS membrane. (a)C1s Deconvolution of PA Membrane XPS. (b)C1s Deconvolution of TPA Membrane XPS.

After alkaline treatment, the C-N bond content on the membrane surface decreased from 29.4% to 25.5%, indicating partial hydrolysis of surface amide bonds.

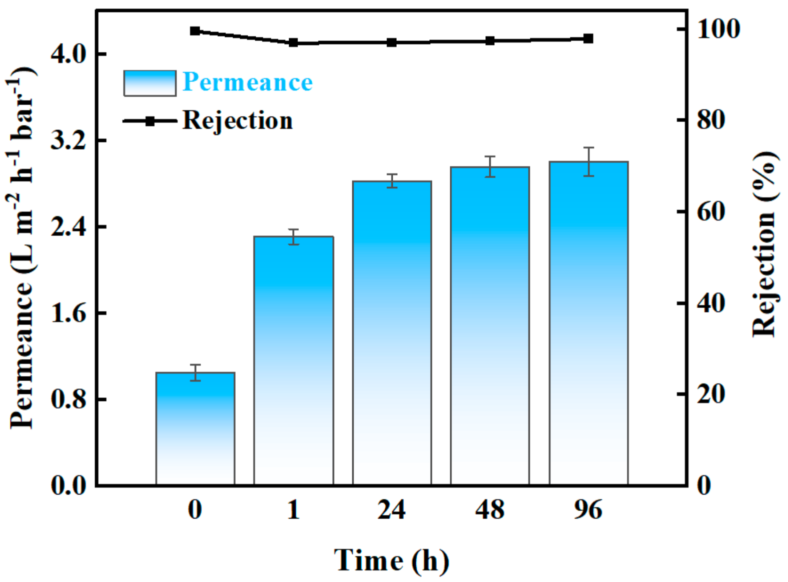

Fig. S5. TPA Membrane Gradient Post-Treatment Time - Performance Variation Chart (soaked in NaOH solution at pH=12.5).

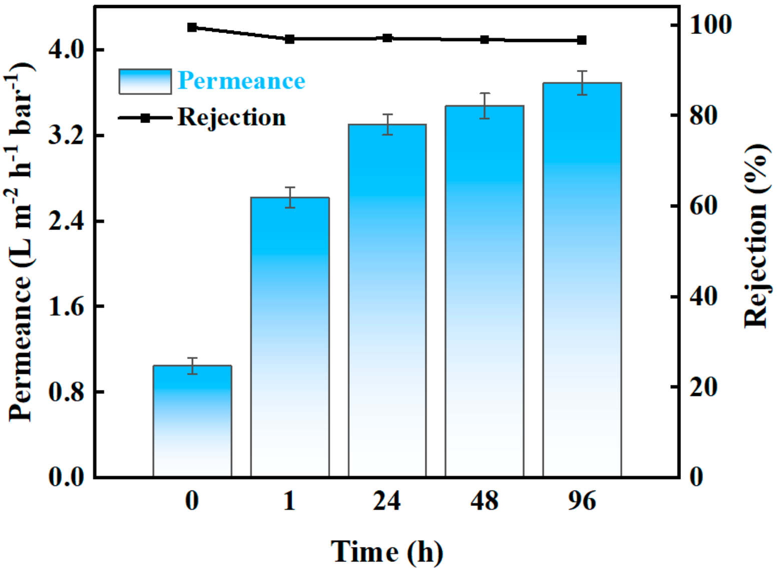

Fig. S6. TPA Membrane Gradient Post-Treatment Time - Performance Variation Chart(soaked in NaOH solution at pH=13.5).

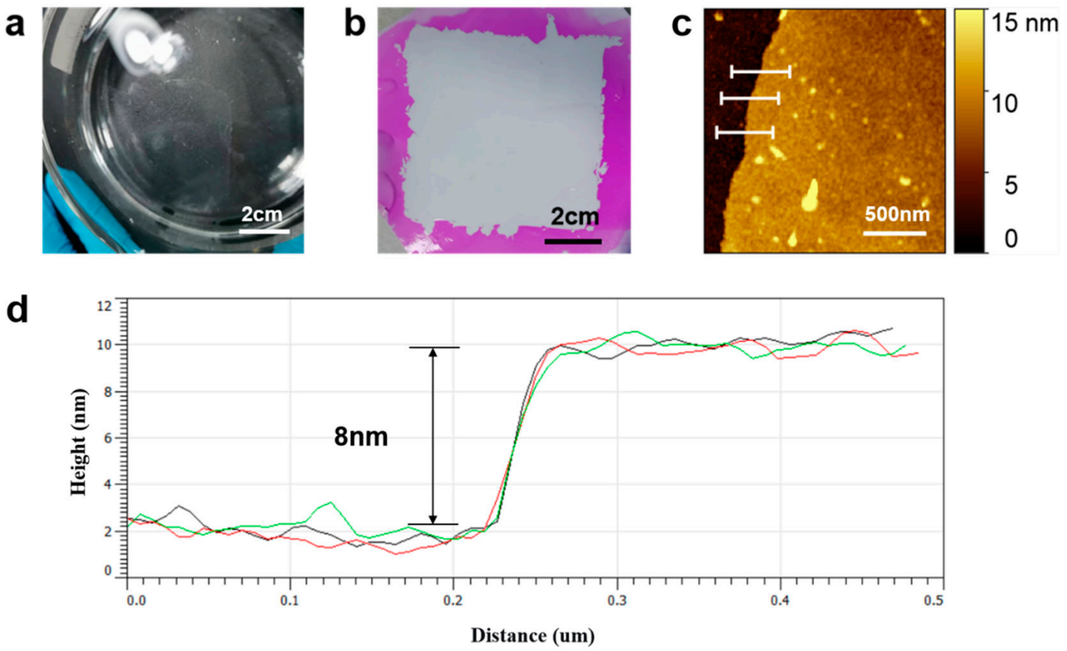

Fig. S7. Morphology and thickness of free-standing PA film. (a)The PA layer floats on the surface of the water. (b)The RO composite membrane, formed by laminating the PA layer onto the Psf substrate membrane, was tested with Rhodamine B dye. The areas with the PA layer did not absorb the dye and appeared to be defect-free. (c)AFM image of the PA membrane supported by a silicon wafer. (d)PA membrane thickness obtained by AFM.

An aqueous solution of MPD (2.5 wt%) and an organic solution of TMC (0.25 wt% in n-hexane) were prepared. A glass petri dish (10×5 cm) was used as the reaction vessel, and a clean silicon wafer (6×6 cm) was placed at the bottom.30 mL of the aqueous MPD solution was poured into the petri dish, followed by slow dropwise addition of 5 mL of the organic TMC solution. Immediately upon addition, a thin polyamide film formed rapidly at the liquid-liquid interface. After 30 s of reaction, the silicon wafer was carefully lifted from the bottom using tweezers; a polyamide film was found to be covering the wafer.The silicon wafer was gently rinsed with n-hexane to remove residual TMC, then immersed in a container filled with deionized water. The polyamide film spontaneously detached from the wafer and floated on the water surface. The floating film was then transferred onto a PSf support by gently lifting it from the water

surface, forming the PA composite membrane.Finally, the PA compo- site membrane was heat-treated in an oven at 70° C for 5 min to enhance the adhesion between the polyamide film and the Psf.

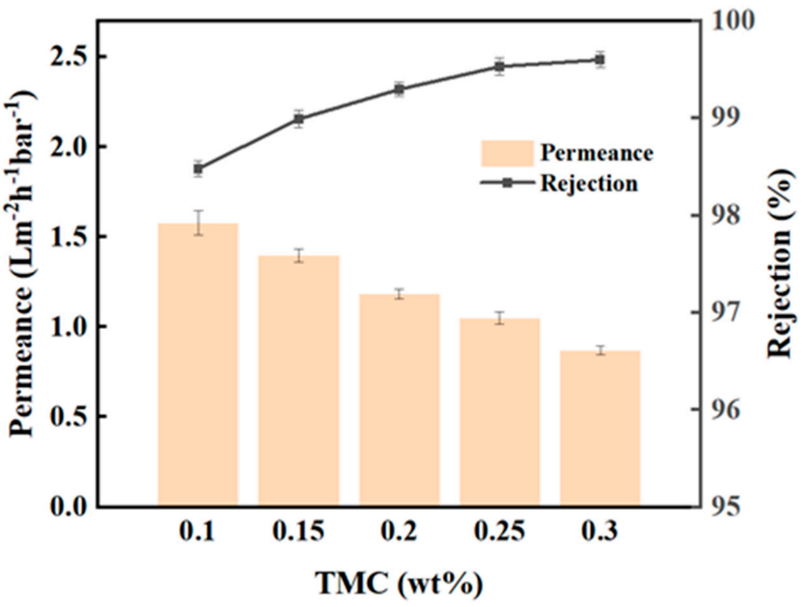

Fig. S8. Effect of TMC Concentration on Membrane Performance.

Although a TMC concentration of 0.10 wt% did yield a continuous selective layer, the NaCl rejection (98.4%) did not meet the desired target. When the TMC concentration was increased to 0.25 wt%, the resulting selective layer exhibited an excellent NaCl rejection of 99.53%, while maintaining an acceptable water permeance. Further increasing the concentration to 0.30 wt% led to a continuous decline in water permeance, accompanied by only a marginal improvement in NaCl rejection.

Compared with the commonly used TMC concentration of 0.10 wt%, a slightly higher concentration facilitates more sufficient reaction with MPD at the interface, promoting the formation of a denser and defect-free membrane layer. Considering that alkali post-treatment slightly reduces the salt rejection rate, based on the performance trade-offs under the conditions of this experiment, we selected 0.25 wt% as the optimal concentration.

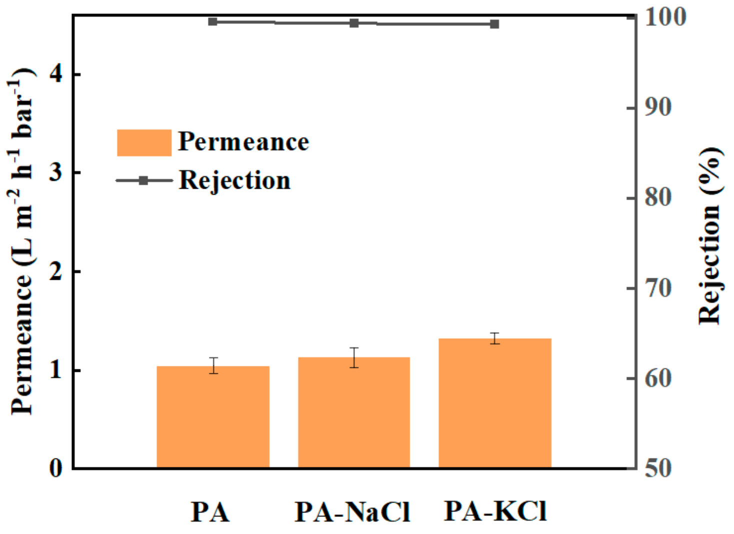

Fig. S9. Effect of Post-Treatment with Na^+ and K^+ Solutions on PA.

Compared with membranes treated with Na^+, membranes treated with K^+ exhibit slightly higher water flux and slightly lower salt rejection, which may be attributed to the differences in the hydrated radii and hydration energies of K^+ and Na^+.

Table S1. The XPS results of the PA and TPA nanofilms.

Nanofilm	C（%）	N（%）	O（%）	N/O
PA	74.73	11.36	13.91	0.81
TPA	71.03	10.34	18.63	0.56

The cross-linking degree (D, %) was calculated via the N/O using the following equations:

$$N/O = \frac{3m+2n}{3m+4n} \quad \text{(q1)}$$

$$D = \frac{m}{m+n} \times 100\%, \quad m+n=1 \quad \text{(q2)}$$

where m and n represent the percentage of the cross-linked and the linear part of the PA matrix, respectively.

It is worth noting that this method provides a simplified estimation rather than an absolute measurement of the degree of crosslinking. A more accurate determination of the degree of crosslinking can be obtained through carboxyl titration or nuclear magnetic resonance of the dissolved polyamide layer.

Table S2. Spherical neutral solutes with various molecular weights were selected for

rejection tests.

Spherical solutes	Molecular structure	Molecular weight (Da)	Stoke radius (nm)[7]	Rejection (%)	
				PA	TPA
Methanol	CH_3OH	32	0.181	20.35	7.47
Ethanol	C_2H_6O	46	0.209	61.66	27.59
Ethylene glycol	$C_2H_6O_2$	62	0.235	76.88	46.91
Triethylene Glycol	$C_6H_{14}O_4$	150	0.334	93.43	86.87
Polyethylene glycol	$HO\ (CH_2CH_2O)_nH$	300	0.439	94.16	93.36
Polyethylene glycol	$HO\ (CH_2CH_2O)_nH$	400	0.491	94.32	93.67

Table S3. Effect of alkaline post-treatment on PA membranes prepared by the free

interface(FIP) method. Each data point is measured using three samples and the average is taken.

Membrane	Alkalinity	Soaking Time (h)	Water permeance (L m^{-2} h^{-1} bar^{-1})	NaCl rejection (%)
Pristine	None	0	0.86	99.10
FIP RO1	12	3	1.39	98.36
FIP RO2	12	8	1.47	98.19
FIP RO3	12.5	3	1.53	98.02
FIP RO4	12.5	8	1.60	97.76
FIP RO5	13	1	1.55	97.89
FIP RO6	13	3	1.75	97.41
FIP RO7	13.5	1	1.69	97.59
FIP RO8	13.5	3	1.81	97.27
FIP RO9	14	1	1.77	97.42
FIP RO10	14	3	1.90	97.15

With the increase in alkali concentration and the extension of soaking time, the water permeability of the membrane shows an increasing trend, while the NaCl rejection rate slightly decreases, which is generally consistent with the post-treatment trend of interfacially polymerized membranes.

Table S4. Performance of RO membranes prepared in this work and in the literature.

Membrane	Operating pressure (bar)	Water permeance ($L\ m^{-2}\ h^{-1}\ bar^{-1}$)	NaCl rejection (%)	Ref.
PA-SMPTES	15.5	2.69	99.29	[56]
TFC-TA/Ag1	15	1.37	97.83	[57]
PA-DMSO/DMAP	15.5	3.95	98.47	[58]
TFC_5-N	15.5	3.49	97.7	[59]
PA-Fe(Ⅱ)/PDS	14	3.25	97.8	[60]
PA-Arg5	16	3.96	98.08	[61]
PA-kGy	15.5	2.08	97.7	[62]
PA-Ngoqd	15	2.66	96.2	[63]
TFC-TPA	15.5	3.21	98.5	This work
Pristine	15.5	1.06	99.5	

Table S5. Performance of RO membranes prepared in this work and in the literature.

Membrane	Operating pressure (bar)	Water permeance (L m^{-2} h^{-1} bar^{-1})	NaCl rejection (%)	Ref.
TFC-GO-ZnS-S	20	1.57	96.3	[64]
TFN-$Ti_3C_2T_x$	20	2.4	96	[65]
TFC-Ag_2S	17	1.9	98.22	[66]
TFC-HBPAC	15	3.02	98.9	[67]
TFC-CON	6	2.2	97.7	[68]
TFC-PK	20	0.6	98	[69]
TFC-H_2	20	2.63	97.9	[70]
TFC-PE	9	1.3	99.3	[71]
TFC-Ag_{20}	20	2.5	99.1	[72]
TFC-DEP	15	2.85	98.2	[73]
TFC-HT	20	3.15	97.3	[74]
TFC-DMI	15.5	3.7	98.36	[50]
TFC-GO	20	1.59	98.7	[75]
TFC-NIPS	20	1.41	98.09	[76]
TFC-PANI	15.5	1.68	99.4	[16]
TFC-PIPC	20	1.87	98.59	[77]
TFC-PEGDA	20	1.82	96.8	[78]
TFN-BTESE	15	1.37	97.2	[79]
TFC-TPA	15.5	3.21	98.5	This work
Pristine	15.5	1.06	99.5	

Table S6. Surface charge density and Donnan potential calculated from zeta potentials

measured in 1 mM KCl solution.

Membrane	Zeta potential (mV)	Surface charge density,σ(mC/m²)	Donnan potential, ψ_D (mV)
PA	-10.24	-0.0234	-24.1
TPA1	-15.68	-0.0362	-30.8
TPA2	-18.62	-0.0432	-33.5

The Surface charge density(σ, C/m^2) and Donnan potential (ψ_D, V) were calculated via the following equations:

$$\sigma = \sqrt{8\varepsilon_r\varepsilon_0 RTC} \cdot \sinh\left(\frac{zF\zeta}{2RT}\right) \qquad \text{(q1)}$$

Where σ and ε_r are the surface charge density (C/m^2) and relative permittivity of the solution (about 80 for water) ; ε_0 is the vacuum permittivity (8.854×10^{-12} F/m); R represents the gas constant (8.314 J/(mol • K)); and R represents the absolute temperature (K, usually 298 K); C is the electrolyte concentration (mol/m^3 , 1 mM = 1 mol/m^3)； z is the ion valence; F is the faraday constant (96485 C/mol); ζ is the zeta potential (V).

$$\psi_D = \frac{RT}{F}\ln\left(\frac{C_{\text{fixed}} + \sqrt{C_{\text{fixed}}^2 + 4C_S^2}}{2C_S}\right), \quad C_{\text{fixed}} = \frac{\sigma \cdot A}{F \cdot V_{\text{pore}}} \qquad \text{(q2)}$$

Where ψ_D is the donnan potential (V); C_{fixed} is the in-membrane fixed charge concentration (mol/m^3); C_S represents the external solution concentration. Estimation of the specific surface area of a typical porous membrane A/V_{pore}≈5×10^6 m^2 /m^3 .

The formation of processed carboxyl groups (-COO$^-$) increased the concentration

of fixed negative charges within the membrane, raising the absolute value of the zeta potential from 24.1 mV to 33.5 mV, indicating a significant enhancement in the membrane's electrostatic repulsion against anions.

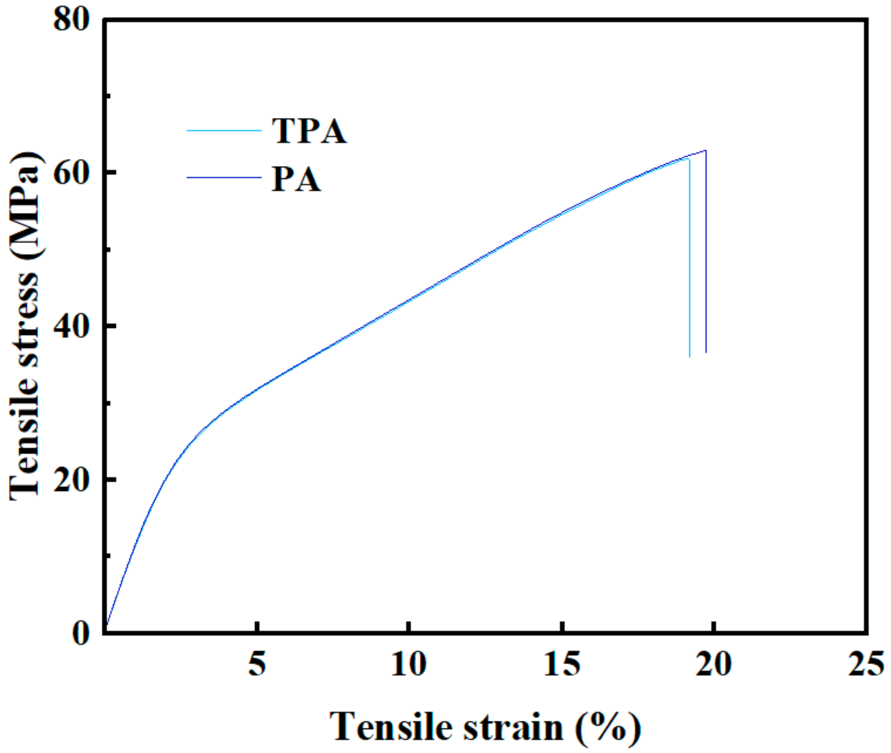

Fig. S10. Tensile Stress-Strain Curves of PA and TPA.

The results showed that the tensile strength and elongation at break of the pristine membrane were 62.94 MPa and 19.71%, respectively, while those of the treated membrane were 61.91 MPa and 19.16%, with only -1.6% and -2.8% variations. It was found that the tensile strength and elongation at break of the TPA membrane only showed slight fluctuations and remain basically consistent with those of the PA membrane. On the one hand, only limited and mild hydrolysis occurs in the polyamide selective layer, which does not cause significant damage to the polymer network, consistent with the observation that the rejection rate does not decrease significantly. On the other hand, the polysulfone support layer (with a much greater thickness than the polyamide layer) exhibits excellent alkali resistance (can withstand 50% NaOH), which effectively maintains the overall structural stability.
